# Standardized Protocol for Chest Tube Management for Trauma Patients Significantly Decreases Complications

**DOI:** 10.1155/2023/2615557

**Published:** 2023-09-21

**Authors:** Christopher A. Dai, Christopher J. Fang, David Schwartz, Jessica Enderson, Ashley McMann, Russel Hyde, Nathan Smith, Jennifer Serfin

**Affiliations:** Department of General Surgery, Good Samaritan Regional Medical Center, 3600 NW Samaritan Dr, Corvallis, OR 97330, USA

## Abstract

**Background:**

As health care shifts to a value-based model with a focus on patient outcomes per dollar spent, it is important to develop and evaluate standardized protocols that ultimately lead to improved patient outcomes and decreased hospital complications. Prior to our chest tube protocol, chest tube management at our Trauma Center was nonuniform and surgeon-specific. The aim of this study was to (1) develop an institutional standardized protocol for chest tube management at our Level II Trauma Center and (2) compare patient outcomes before and after the implementation of our protocol.

**Methods:**

An institutional, standardized protocol was initiated at our Level II-Certified Trauma Center teaching hospital in 2014. An IRB-approved, single-institution retrospective chart review was performed between January 2011 and May 2017, in order to capture the 3 years prior and 3 years after protocol implementation. All patients with a diagnosis of hemothorax or pneumothorax (H/PTX) from blunt or penetrating trauma that resulted in a >24 French chest tube placement were included in the study. Patients were excluded if interventional radiology (IR) placed the chest tube, the mechanism was nontraumatic, or the patient expired at index hospitalization. Univariate analyses were performed to evaluate significant differences in patient outcomes before and after the implementation of the protocol.

**Results:**

A total of 143 patients were analyzed for this study, with 43 preprotocol patients and 100 postprotocol patients. Hospital length of stay (LOS), persistent H/PTX, and the need for further surgical intervention all improved after the implementation of the standardized protocol (*p* < 0.04).

**Conclusions:**

Our standardized protocol for chest tube management at our Level II Trauma Center significantly improved patient outcomes and can serve as a model for similar institutions.

## 1. Introduction

Thoracic trauma is a common and significant injury pattern with serious potential consequences, resulting in 25% of the 140,000 deaths per year in trauma patients in the United States [1–3]. Up to 75% of thoracic traumas can be managed with a thoracostomy tube or chest tube and volume resuscitation [2, 4]. However, these patients are at an increased risk for mortality and long-term morbidity, so multidisciplinary care and coordination is essential in achieving high-quality outcomes in these patients [2, 5].

Limited data exist on the standardized management for patients with hemothorax or pneumothorax (H/PTX) following blunt or penetrating trauma injuries. It is not uncommon for hospitals and general surgeons to approach chest tube management in an individual and nonuniform manner. Prior to the implementation of a new, standardized approach to chest tube management, our hospital chest tube management was at the discretion and preferences of the attending surgeon on call. This led to uncoordinated treatment plans with shift changes and patient hand-offs. With an increased emphasis on improving patient outcomes with a healthcare model shift to value-based health care, developing standardized models for treatment protocols can be an important and implementable route for hospitals to undertake [6]. Studies have begun to emerge describing standardized protocols for chest tube insertion and management in order to improve the quality of care involved in these patients [1, 7, 8]. Martin et al. described an algorithmic approach to thoracostomy tube management for their Level 1 Trauma Center patients [8]. These algorithms lead to an organized approach in treating chest tube patients and were used to develop our institutional framework for clinical management of this patient population.

Therefore, the aims of this study were (1) to develop an institutional standardized protocol for chest tube management at our Level II Trauma Center and (2) compare patient outcomes before and after the implementation of our protocol. Our hypothesis is that the standardized treatment of trauma patients undergoing chest tube management for blunt or penetrating trauma will lead to improved outcomes and decreased hospital complications.

## 2. Methods

### 2.1. Study Design

After the institutional review board's (IRB) approval was obtained, we retrospectively analyzed chart data for trauma patients who underwent chest tube placement for hemothorax or pneumothorax (H/PTX) from blunt or penetrating chest trauma between January 2011 and May 2017 at our Level II-certified Trauma Center teaching hospital. This time frame was selected to capture the preceding and following three years of protocol implementation. Patient cohort preceding the protocol was labeled as “precohort,” and following the protocol was labeled “postcohort.” All included patients had placement of a >24 French chest tube for H/PTX, with larger H/PTX requiring larger bore catheters. Patients who underwent chest tube placement by the interventional radiology (IR), pigtail catheters, from a nontraumatic mechanism, or expired during the index admission were excluded from this study. Patient-specific demographics included age, gender, and mechanism of injury. Patient outcomes investigated included average hospital length of stay (LOS), persistent H/PTX, recurrent H/PTX, and the need for further surgical intervention during hospital admission.

### 2.2. Standardized Chest Tube Management Protocol Development

**Figure 1:**
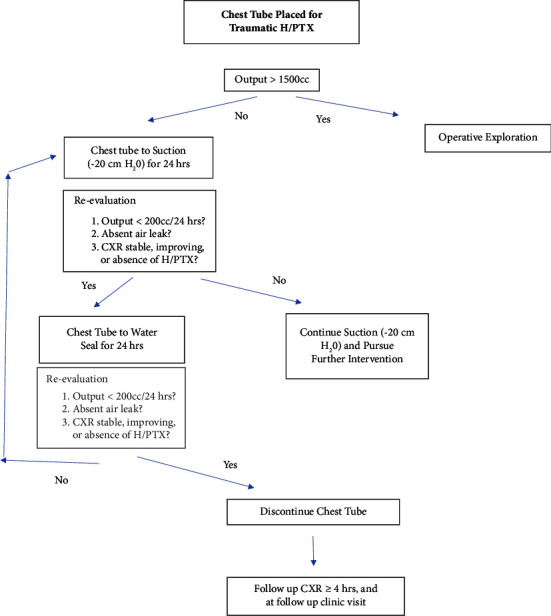
Chest tube management algorithm.

Our algorithmic approach design is shown in Figure 1. The protocol was designed based on the reported chest tube algorithms, with institution-specific modifications at our Level II Trauma Center [1, 8, 9]. The algorithm is described as follows. Chest tubes were placed for appropriate patients who sustained traumatic H/PTX with an index chest X-ray performed at the time of insertion. If immediate output was over 1,500 mL, patients were taken to the operating room (OR). Chest tubes were placed to wall-mounted vacuum at −20 cm H_2_O for a 24-hour period. After the 24-hour period elapsed, patient was re-evaluated with CXR. If repeat CXR showed worsening of H/PTX, the output was over 200 mL over 24 hours, or air leak was present—the patient was kept on continuous suction and further interventions were pursued [9]. If none of these conditions were present, the patient was placed on water seal for 24 hours. After the 48-hour period elapsed, if worsening of H/PTX continued, the output was over 200 mL over 24 hours, or air leak was present— patient would return to the continuous suction with re-evaluation in 24 hours. If none of these conditions were present, the chest tube was discontinued with a follow-up CXR in 4 hours and at follow-up clinic visit.

### 2.3. Statistical Analysis

Mann–Whitney *U*-tests and Fisher's exact tests were used where appropriate to compare the outcomes in patients who received treatment before and after the implementation of the standardized protocol. The odds ratios were also calculated for patient outcomes. All statistical analyses were performed using SAS v9.4 (SAS Institute, Cary, NC). Statistical significance was defined as *p* < 0.05.

## 3. Results

A total of 143 patients were included in this study, with 43 patients comprising the precohort, and 100 patients comprising the postcohort (Table 1). Patient demographics are shown in Table 1. Age, sex, and mechanism of trauma were similar between groups (Table 1).

**Figure 2:**
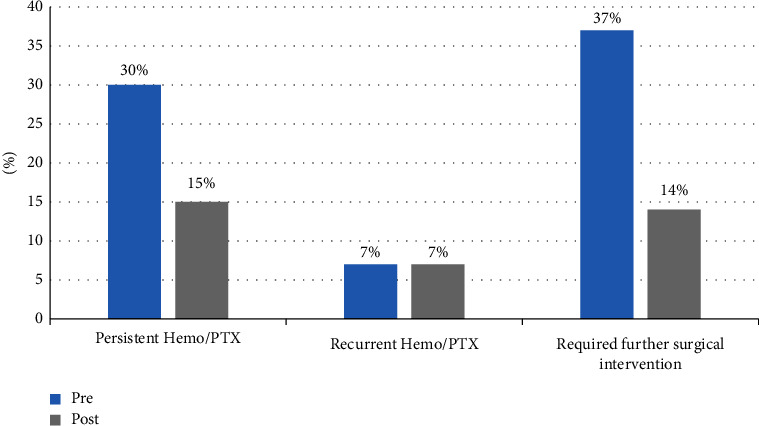
Bar graph displaying comparisons for patient outcomes in the precohort and postcohort for the chest tube management protocol.

Hospital LOS significantly decreased in the postcohort as compared to the precohort (*p* = 0.02), with the postcohort LOS shorter by an average of 2 days (Table 2). Persistent H/PTX rates were 15% lower in the postcohort (*p* = 0.04). The odds of persistent H/PTX were estimated to be 0.40 times lower (or 60% lower) after the protocol was implemented (OR = 0.40, 95% CI = 0.17–0.95). Recurrent H/PTX rates were identical (7%). Only 14% of the postcohort needed further surgical intervention vs 37% in the precohort (*p* = 0.003; Figure 2). The odds of needing further surgical intervention are estimated to be 0.27 times lower (or 73% lower) after the protocol was implemented (OR = 0.27; 95% CI = 0.12–0.63).

## 4. Discussion

Our study found that after the implementation of a standardized, algorithmic approach to chest tube management for patients sustaining a traumatic H/PTX resulted in a significant improvement in patient outcomes and hospital complications. Hospital LOS decreased by an average of 2 days, persistent H/PTX rates halved, and surgical intervention rates decreased by 23%. In our precohort, there was a complication rate of 37% (16/43), and our postcohort had a complication rate of 22% (22/100). Our improved complication rate seen was comparable to a previous study by Menger et al. that reported a thoracostomy tube complication rate of 22.1% following thoracic trauma [10].

Recently, the Western Trauma Association developed a critical decisions algorithm for the evaluation and management of traumatic pneumothorax [11]. Their recommendations were based on the available published studies and expert panel opinion. They found considerable research gaps in the published literature including differentiation in the management of blunt vs penetrating mechanism and physiological impact of size of the PTX. They concluded that pneumothoraces require an objective view to management and demand ongoing investigations [11]. Our study adds to the limited body of literature to guide practitioners with a systematic, algorithmic approach.

Our hospital LOS improved to an average of 9.7 days. This remains a higher LOS when compared to the previous reports of 4.1 days in similar cohorts [9]. This difference may be attributed to a higher level of injury severity and is comparable to a Level 1 Trauma Center study by Martin et al. with an average LOS of 10.4 days [8]. The high standard deviation we saw in our cohort, with outliers in the higher range, may have positively skewed our data. Hospital duration may also be affected by concomitant injuries sustained by the studied patient population.

Our study found no differences in the recurrent H/PTX, but found a significant improvement in persistent H/PTX. This can theoretically be attributed to strict algorithmic protocol guidelines in which patients are immediately placed on water seal at a standardized pressure. Patients requiring surgical intervention at any time point also decreased, which we believe was due to vigilant re-evaluation check points with limited indications for OR management. As these injuries are generally recommended to be treated expectantly, a decrease in OR utilization is an important outcome in this patient population.

The strengths of the current study include description of a single-institution experience with the implementation of a new, standardized chest tube management protocol. While there is a general consensus on chest tube management, there is limited evidence on algorithms and management postinsertion. The present study contains several limitations. All cases were from a single, Level II Trauma Center teaching hospital, which could limit generalizability. This was a retrospective study which inherently creates concerns for the selection bias and confounding. It also cannot determine the causation and only imply that the improvement in outcomes was associated with implementing practice guidelines. Furthermore, our sample sizes are relatively small and are not in equal proportions. In line with this, we were unable to delineate between the differences in our hemothorax and pneumothorax patients due to our sample size and type 2 error probability. The difference in the management of hemothorax and pneumothorax (e.g., chest tube size) could represent a potential bias of our study. However, standardized protocols for chest tube management are limited [1, 8]—so we believe our study represents an important model. Lastly, variability exists among institutions; resource constraints and patient-specific factors may require alternative approaches on a case-by-case manner. Despite these limitations, our data did show decreased LOS and less surgical intervention in patients with traumatic H/PTX when their chest tube was managed using uniform practice guidelines.

## 5. Conclusion

In conclusion, our standardized algorithmic protocol for chest tube management of blunt or penetrating trauma patients sustaining a hemothorax or pneumothorax resulted in decreased hospital complications. Continued efforts towards improving patient outcomes and decreasing complications for trauma patients are paramount for improving healthcare delivery. The systematic approach described here can be modeled at similar institutions for enhanced patient care.

## Figures and Tables

**Table 1:** Patient demographics.

	Precohort (*n* = 43)	Postcohort (*n* = 100)	*p* value
Age in years (SD) Min-Max^*∗*^^†^	48 (17) 18–91	51 (21) 17–97	0.45
Male gender^‡^	58% (25)	70% (70)	0.18
Blunt mechanism^‡^	91% (39)	95% (95)	0.45

SD = standard deviation. ^†^Mann–Whitney *U*-tests were used to compare quantitative variables, and ^‡^Fisher's exact tests were used to compare the categorized variables across all groups. ^*∗*^These values are given as the mean and standard deviation, with a range of minimum to maximum values. The remaining values are given as a percentage of the specific category. Gender options included male or female. Mechanisms included blunt or penetrating.

**Table 2:** Patient outcomes comparing precohort and postcohort of the chest tube management protocol.

	Precohort (*n* = 43)	Postcohort (*n* = 100)	*p* value
Hospital LOS (SD) Min-Max^†^^*∗*^	11.6 (9.9) 2–45	9.7 (10.5) 1–50	0.02
Persistent H/PTX^‡^	30% (13)	15% (15)	0.04
Recurrent H/PTX^‡^	7% (3)	7% (7)	0.99
Required further surgical intervention^‡^	37% (16)	14% (14)	0.003

LOS = length of stay; SD = standard deviation; H/PTX = hemothorax or pneumothorax. ^†^Mann–Whitney *U*-tests were used to compare quantitative variables, and ^‡^Fisher's exact tests were used to compare the categorized variables across all groups. ^*∗*^These values are given as the mean and standard deviation, with a range of minimum to maximum values. The remaining values are given as a percentage of the specific category.

## Data Availability

The data used to support the findings of this study are available from the corresponding author upon request.
